# Formal and informal prediction of recurrent stroke and myocardial infarction after stroke: a systematic review and evaluation of clinical prediction models in a new cohort

**DOI:** 10.1186/1741-7015-12-58

**Published:** 2014-04-04

**Authors:** Douglas D Thompson, Gordon D Murray, Martin Dennis, Cathie LM Sudlow, William N Whiteley

**Affiliations:** 1 Edinburgh MRC Hub for Trials Methodology Research, Centre for Population Health Sciences, University of Edinburgh Medical School, Teviot Place, Edinburgh EH8 9AG, UK; 2 Division of Clinical Neurosciences, University of Edinburgh, Bramwell Dott Building, Western General Hospital, Edinburgh EH4 2XU, UK

**Keywords:** Systematic review, Meta-analysis, Stroke, Prediction, Statistical modelling, Evaluation, Development

## Abstract

**Background:**

The objective of this study was to: (1) systematically review the reporting and methods used in the development of clinical prediction models for recurrent stroke or myocardial infarction (MI) after ischemic stroke; (2) to meta-analyze their external performance; and (3) to compare clinical prediction models to informal clinicians’ prediction in the Edinburgh Stroke Study (ESS).

**Methods:**

We searched Medline, EMBASE, reference lists and forward citations of relevant articles from 1980 to 19 April 2013. We included articles which developed multivariable clinical prediction models for the prediction of recurrent stroke and/or MI following ischemic stroke. We extracted information to assess aspects of model development as well as metrics of performance to determine predictive ability. Model quality was assessed against a pre-defined set of criteria. We used random-effects meta-analysis to pool performance metrics.

**Results:**

We identified twelve model development studies and eleven evaluation studies. Investigators often did not report effective sample size, regression coefficients, handling of missing data; typically categorized continuous predictors; and used data dependent methods to build models. A meta-analysis of the area under the receiver operating characteristic curve (AUROCC) was possible for the Essen Stroke Risk Score (ESRS) and for the Stroke Prognosis Instrument II (SPI-II); the pooled AUROCCs were 0.60 (95% CI 0.59 to 0.62) and 0.62 (95% CI 0.60 to 0.64), respectively. An evaluation among minor stroke patients in the ESS demonstrated that clinicians discriminated poorly between those with and those without recurrent events and that this was similar to clinical prediction models.

**Conclusions:**

The available models for recurrent stroke discriminate poorly between patients with and without a recurrent stroke or MI after stroke. Models had a similar discrimination to informal clinicians' predictions. Formal prediction may be improved by addressing commonly encountered methodological problems.

## Background

About a quarter of the patients who survive their stroke have a recurrent stroke within five years [1]. Any method that could reliably discriminate between those patients at high risk and those at low risk of recurrent stroke would be useful. Patients and their clinicians might use such information to make decisions about different preventive strategies and better target resources.

Clinical prediction models (also known as prognostic/statistical models or scores) combine multiple risk factors to estimate the absolute risk of a future clinical event. No estimate is perfect, but a model that predicted the risk of recurrent stroke just as well as or better than an experienced clinician might improve clinical practice. Some prediction models are used widely in clinical practice to quantify risk of future vascular events (for example, the ASSIGN [2], Framingham [3], and CHADS [4] scores). None of the prediction models for recurrent events after stroke is in widespread use, either because their statistical performance is too poor or because the models are too hard to use.

We sought to pool measures of statistical performance of existing models and investigate whether there were aspects of study design or analysis that might be improved in the development of new models. Therefore, we systematically reviewed the literature on the development and evaluation of prediction models for recurrent vascular events after ischemic stroke in order to assess: (1) the quality of the cohorts and the statistical methods used in their development; and (2) their external performance. We aimed to compare clinical prediction models with clinicians’ informal predictions in a new prospective cohort study.

## Methods

The analysis protocol is available at [5]. We searched Medline and EMBASE databases from 1980 to 19 April 2013 with an electronic search strategy using a search term for ‘stroke’ and synonyms for ‘clinical prediction models’ [see Additional file 1] [6,7]. We also searched reference lists, personal files and Google Scholar [8] for citations of relevant articles.

### Inclusion criteria

Eligible articles developed and/or evaluated a multivariable clinical prediction model for the risk of recurrent ischemic stroke, myocardial infarction (MI) or all vaso-occlusive arterial events in cohorts of adult patients with ischemic stroke (or mixed cohorts of ischemic stroke and transient ischemic attack (TIA). We excluded any studies using cohorts that included hemorrhagic strokes. We made no language restrictions.

### Data extraction

One author (DDT) screened all titles and abstracts identified by the electronic search against the inclusion criteria prior to full text assessment. Two authors (DDT and WNW) extracted data independently with a detailed data extraction form developed and piloted by three of the authors (DDT, GDM and WNW). We resolved discrepancies by discussion. We adapted quality items from similar systematic reviews [6,7,9-13] (Table 1) as no recommended tool for the appraisal of quality of prediction models currently exists. We distinguished two types of articles: (1) development studies reporting the construction of a prediction model, and (2) evaluation studies (also known as validation studies) assessing model performance in a cohort of new patients.

**Table 1:** Quality assessment of articles

**Quality item**	**Comment**
**Internal validity**	
*Sample cohort*	Prospectively collected data are of greater quality than retrospectively collected data and are preferred for model development [14].
*Loss to follow up*	Loss to follow up is common. Investigators should state the number of patients lost (or else the completeness of follow-up [15] which takes into account the duration of follow-up) along with reasons/explanations. An arbitrary proportion thought adequate for analysis is 90% complete follow-up [7].
*Predictive/outcome variables*	Predictors and outcomes/follow-up time should be explicitly defined: otherwise invalid predictions may be produced.
*Missing values*	A transparent summary of missing data and the methods used to handle them should be provided. Complete-case analysis should be avoided in favor of multiple imputation methods [16,17]. A general rule of thumb suggests that imputation should be considered if the proportion of missingness exceeds 5% of the data [18].
**Statistical validity**	
*Model building strategy*	*A priori* clinical knowledge should be used to inform selection of risk factors. Data driven predictor selection (for example, stepwise selection) should be avoided where possible [19,20].
*Handling of continuous variables*	Arbitrary categorization should be avoided [21]. Defined cut-points must be based on clinical reasoning.
*Sample size*	The sample size used in derivation (derivation sample) must be reported along with a sufficient description of baseline characteristics. The number of patients with the outcome event in follow-up (effective sample size) should be reported: 10 events per fitted parameter is often used as a minimum number [22].
**Model evaluation**	
*Evaluation*	Internal validation techniques (for example, bootstrap sampling or cross-validation) provide a minimum check of overfitting and optimism. External evaluation in new data is the most rigorous assessment of model generalizability.
*Description of external cohort*	A description of the baseline characteristics should be reported to enable a comparison of the validation cohort to the development cohort.
*Discrimination and calibration*	Discrimination metrics should be provided, for example, the area under the receiver operating characteristic curve (AUROCC). Model calibration should be studied using a calibration plot with estimated slope and intercept provided.

All measures of model performance were extracted along with any associated measures of uncertainty (for example, 95% confidence intervals (CI) or standard error). Two commonly used measures of performance are: ‘calibration’ and ‘discrimination’ [23]. Calibration summarizes how well the observed events match the predicted events by dividing the cohort into groups of predicted risk (for example, quintiles or deciles) and comparing the mean predicted risk with the observed frequency. Discrimination summarizes how well a model separates patients with the event in follow-up from those without. The *c*-statistic is a commonly used rank order measure of discrimination ranging from no better than chance (0.5) to perfect (1.0) discrimination. For a given pair of patients, one with the event of interest and one without, the *c*-statistic is interpreted as the probability that a greater predicted risk is given to the patient with the event than the patient without. In logistic regression the Area under the Receiver Operating Characteristic Curve (AUROCC) is equivalent to the *c*-statistic.

### Meta-analysis

If three or more studies assessed a model’s performance we performed a random-effects meta-analysis using the DerSimonian and Laird method [24] (implemented with the ‘metafor’ package [25] in R version 2.13.1). A random-effects meta-analysis allows for differences in model performance that may be explained by differing case mix between studies (for example, older patients or more severe baseline strokes and so on). We estimated the 95% prediction interval (PI) associated with the individual pooled estimates which differs somewhat from the CI [26]. The CI summarizes the precision of a parameter estimate whereas the PI provides a plausible range within which an unknown estimate will be expected to lie in 95% of future samples. We assessed publication bias with Contour-enhanced funnel plots [27]. The PRISMA checklist for our review is available as an online supplement [see Additional file 1].

### Evaluation cohort

Evaluation in an external cohort is the most robust test of model performance and generalizability. The Edinburgh Stroke Study (ESS) was a prospective observational study of stroke patients admitted to the Western General Hospital in Edinburgh between April 2002 and May 2005 with a minimum follow-up of one year. Details on the study’s design are available elsewhere [28]. Clinicians were asked to use ‘gut-feeling’ to estimate the absolute risk of a recurrent stroke or a vascular event (that is, stroke, MI or vascular death) within one year in patients seen as outpatients. We compared models we identified using measures of discrimination and calibration to clinicians’ informal estimations.

## Results

**Figure 1:**
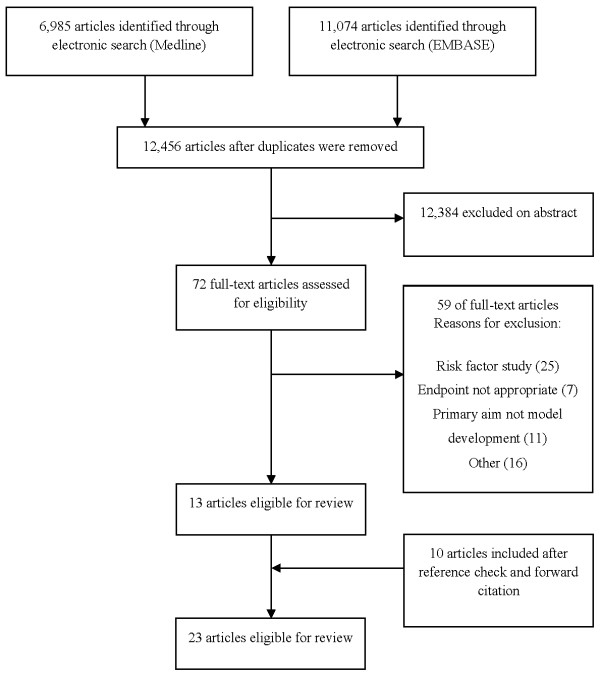
PRISMA flow diagram of selected studies.

**Figure 2:**
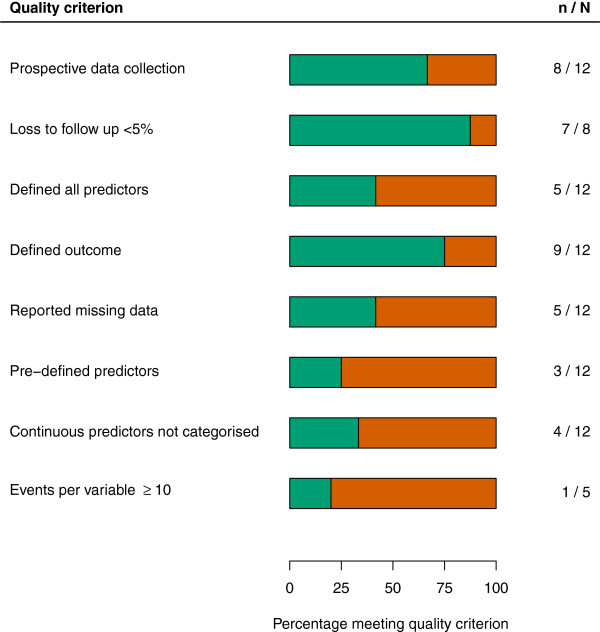
Aspects of model development.

We screened 12,456 articles by title and abstract (PRISMA diagram Figure 1), thirteen of which were eligible for review. A further ten were identified from reference list checks and forward citation searches in Google Scholar. We found twelve development studies [see Additional file 1 and Figure 2] that developed a total of 31 models (a median of two per study, interquartile range (IQR) one to three). We found eleven evaluation studies that evaluated four models [see Additional file 1]. Only one relevant study written in a language other than English was included [29].

### Model development studies: cohort characteristics

Studies which collect data prospectively have a lower risk of information and selection biases for both baseline data and outcome events occurring during follow-up. Most studies used prospectively collected data, although four of twelve did not [30-33], one of which [33] used prospective trial data but included retrospective events obtained beyond the trial’s original follow-up period. Few (four of twelve) development studies recruited patients consecutively from routine practice [30,31,34,35]. Loss of patients to follow-up often occurs when studies last for long time periods. Most (nine of twelve) [30-33,35-39] development studies reported loss to follow up; where this could be calculated (seven of eight) [31-33,35-39] rates of loss were small (less than 5%).

The most frequent variables included in multivariable clinical prediction models were: age, history of TIA or stroke, history of hypertension, and diabetes [see Additional file 1]. Five articles [31,32,34,36,39] defined all predictors, three [30,35,37] defined only some, and four [33,38,40,41] did not define any. Most articles defined outcome adequately, although three did not define the outcome and/or the duration of follow-up [38,40,41].

Missing baseline data occur frequently when collecting information from patients. A complete case analysis using only those patients with complete baseline data risks selection bias and loss of information. Five of the development studies [32-34,38,41] reported missing data, four [32-34,38] of which stated the impact a complete case analysis had on the derivation sample size. No attempts were made to impute missing data.

### Model development studies: statistical methods

Most investigators collect more potential predictors than are included in a final model. Data dependent methods (for example, univariate selection or stepwise selection) are often used to select a few important variables from those available to develop a prediction model. This can lead to over-fitted models that perform over-optimistically in their development datasets which may be impossible to replicate in external evaluation [42]. Most of the studies used data dependent variable selection methods: stepwise selection (two of twelve) [32,35]; univariate significance tests (four of twelve) [30,31,34,36]; and further reduction of univariate selection by inspection of multivariable significance (two of twelve) [33,38]. Three modifications of pre-existing prediction models were identified with new predictors chosen by clinical justification [37,39,41]. One study gave no description of how variables were selected [40].

Internal evaluation methods can use the model development data to provide optimism-corrected estimates of model performance. Few authors internally assessed the performance of their models (three of twelve) using such cross-validation methods [30,31,37].

Models were derived using Cox proportional hazard regression (nine of twelve) [30,32-36,38,39,41] or multivariable binary logistic regression (three of twelve) [31,37,40]. Most studies presented their models as point scores (seven of twelve) by rounding regression coefficients [30-32,34,37,40,41]. The categorization of a continuous predictor results in the loss of information. The majority of studies categorized continuous predictors (eight of twelve) [30-32,34,36,37,40,41], only one of which gave some clinical justification for the cut-points chosen [37]. The remaining four studies used a mixture of categorized and continuous variables [33,35,38,39].

A common rule of thumb used in prediction model literature is the ‘ten events per tested variable’ (10 EPV) rule. The median total sample size across the twelve development studies was 1,132 (IQR 522 to 3,123). Where reported (nine of twelve), the median number of events was 73 (IQR 60 to 102). Only one of the five studies where the EPV could be calculated had more than the minimum recommended EPV [37].

### Model evaluation studies: study characteristics

The ESSEN Stroke Risk Score (ESRS) [40], the Stroke Prognosis Instrument II (SPI-II) [41], the Recurrence Risk Estimator at 90 days (RRE-90) [30] and the Life Long After Cerebral ischemia (LiLAC) [33] were externally evaluated in eleven different studies [see Additional file 1]. We identified four additional evaluations among the model development studies [30,37,39,41] giving fifteen evaluation cohorts: five evaluations of the ESRS [29,37,43-45]; three of the SPI-II [41,46,47]; five head-to-head comparisons of the ESRS and the SPI-II [30,39,48-51]; one head-to-head comparison of the ESRS and the RRE-90 [52]; and one comparing the ESRS, the SPI-II and the LiLAC models [50].

The median sample size in the 15 evaluation cohorts was 1,286 (IQR 619 to 5,004). Various combinations of events and follow-up periods were used yielding 49 specific AUROCC values for extraction [see Additional file 1]. Where the effective sample size could be determined the median size was 86 (IQR 58 to 134).

### Model evaluation studies: statistical performance

**Figure 3:**
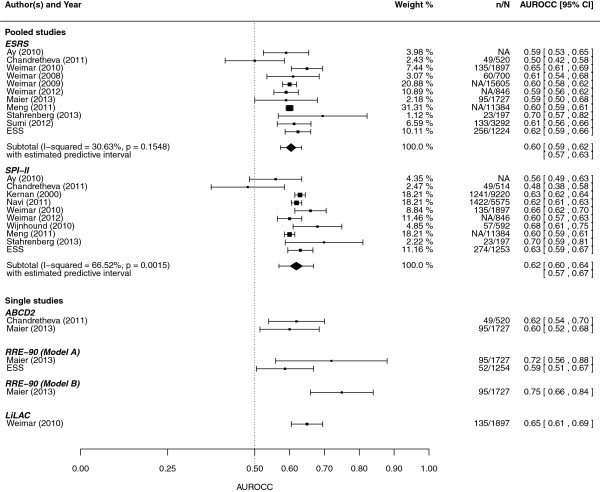
**Meta-analysis of AUROCC values for ESRS and SPI-II (percentage weights are from random effects analysis).** N = sample size, n = number of events in follow-up, and NA missing information. AUROCC, area under the receiver operating characteristic curve; ESRS, ESSEN Stroke Risk Score; SPI-II, Stroke Prognosis Instrument II.

The pooled AUROCC value for the ESRS was 0.60 (95% CI 0.59 to 0.62) and for the SPI-II was 0.62 (95% CI 0.60 to 0.64) (Figure 3). Six head-to-head comparisons of the ESRS and the SPI-II were identified. Four of these [39,49-51] (the other two [30,48] used much shorter follow-up periods) were pooled to calculate the AUROCC estimates: 0.61 (95% CI 0.58 to 0.64) with 95% PI (0.29 to 0.93) and 0.62 (95% CI 0.59 to 0.66) with 95% PI (0.23 to 0.99), respectively, for the ESRS and the SPI-II scores. These findings were robust to sensitivity analyses [see Additional file 1]. One evaluation study for the RRE-90 score estimated an AUROCC of 0.72 (95% CI 0.64 to 0.80) [52] and another of the LiLAC score estimated an AUROCC of 0.65 (95% CI 0.61 to 0.70) [50]. We identified two evaluations of the ABCD2 score [48,52,53]. Although the ABCD2 score was developed and designed for patients with TIA (and, therefore, did not meet our inclusion criteria) its performance was similar to other clinical prediction models for recurrent stroke (Figure 3). Only one study assessed the calibration of the SPI-II score which found it to be good but only after re-calibration [47]. There was no evidence for small study (that is, publication) bias [see Additional file 1].

### Model evaluation: comparative performance with clinical gestalt

Baseline characteristics for the ESS can be found online [see Additional file 1].We were able to evaluate five of twelve models in the ESS (Table 2). In the ESS data, 575 patients had informal predictions for vascular outcomes by one year. We were able to obtain information regarding thirteen of the clinicians making predictions for 542 (94%) of the patients. Of these: eight were neurologists (62%) and five were stroke physicians (38%); seven were in training (54%) and six were fully trained (46%). The median number of patients seen per clinician was seven (ranging from 1 to 217). For recurrent stroke within one year clinicians discriminated poorly between those who did and those who did not suffer an event with an AUROCC of 0.54 (95%CI 0.44 to 0.62). Formal prediction also discriminated poorly with AUROCC measures varying between 0.48 and 0.61. For risk of vascular events, clinicians again discriminated poorly with an AUROCC of 0.56 (95%CI 0.48 to 0.64) and formal prediction ranged from 0.56 to 0.61. The AUROCCs from the ESRS and the SPI-II were calculated for all patients in the ESS for any vascular event and added to the meta-analysis.

**Table 2:** Performance of models in the Edinburgh Stroke Study

	**Recurrent stroke (50/671)**^**a**^			**Any vascular event (80/671)**^**a**^			**Original development outcome**^**b**^		
**Model**	**n/N**	**AUROCC**	**95% CI**	**n/N**	**AUROCC**	**95% CI**	**n/N**	**AUROCC**	**95% CI**
Clinical gestalt	40/575	0.53	0.44 to 0.63	63/574	0.56	0.48 to 0.64	-	-	-
ESRS	50/664	0.56	0.48 to 0.64	80/664	0.57	0.50 to 0.63	101/1,224	0.54	0.49 to 0.60
SPI-II	50/669	0.58	0.49 to 0.66	80/669	0.59	0.52 to 0.66	274/1,253	0.63	0.59 to 0.67
RRE-90^c^	50/671	0.61	0.52 to 0.69	80/671	0.59	0.53 to 0.66	52/1,254	0.59	0.51 to 0.67
Putaala	50/669	0.48	0.39 to 0.57	80/669	0.56	0.49 to 0.63	269/1,247	0.65	0.61 to 0.68
Dhamoon	50/668	0.60	0.52 to 0.68	80/668	0.61	0.54 to 0.67	205/1,253	0.73	0.69 to 0.76

Cell entries are AUROCCs for a recurrent stroke by one year, all vascular events by one year, and the outcome as defined in development. Few patients were deleted due to missingness for prediction models (671 outpatients with 50 strokes in follow-up and 80 vascular events; and for all patients 1,257, 102 and 274 respectively). ^a^Outpatients only; ^b^one year follow-up was available for all patients in the ESS; ^c^Model A was the clinical based model. AUROCC, area under the receiver operating characteristic curve; CI, confidence interval; ESRS, ESSEN Stroke Risk Score; RRE-90, Recurrence Risk Estimator at 90 days; SPI-II, Stroke Prognosis Instrument II.

## Discussion

We found four externally evaluated clinical prediction models for the prediction of recurrent stroke and MI after stroke: the ESRS, the SPI-II, the RRE-90 and LiLAC. The discriminative performances of the models were similar to one another, but only modest at best, with AUROCC values ranging from 0.60 to 0.72. The performance of some of the clinical prediction models although modest was similar to experienced clinicians.

There were some weaknesses in the methodology of model development which may explain the modest performance observed in external evaluation studies of clinical prediction models. First, continuous variables were often categorized which leads to a loss of predictive information. Second, data-dependent variable selection may have led to over-fitting of models to the observed data. Third, cohorts were generally too small for reliable model development: we found only one study with more than the recommended 10 EPV. Small samples can lead to prediction models that are over-fit on the available data which is further compounded by implementing a complete case analysis. Fourth, the cohorts used to develop the models had weaknesses that are frequent in epidemiological studies: there were missing baseline data; whether the recruited patients were representative of those seen in routine clinical practice was uncertain; some data were collected retrospectively; and most cohorts did not record all potentially predictive variables. For example, the presence of multiple infarcts on MR scanning was only considered in one model [30,54].

While it seems more likely that a well-developed model will have better performance in external evaluation, the only reliable method for choosing between models is their performance in evaluation studies of representative patients. Despite the differences in the methods of derivation of the ESRS, the SPI-II and the LiLAC, they discriminated similarly (and modestly) between patients with and without recurrent stroke [50]. The ESRS and the SPI-II have four predictors in common (age, history of TIA or stroke, diabetes and blood pressure). Three head-to-head comparisons demonstrated a relative difference in AUROCC which did not exceed 2% [49-51].

This is one of the few studies of the performance of clinicians’ predicting vascular events. Although such investigations perhaps provide the most robust argument for or against the use of statistical prediction, they remain rare. For example, there are many prediction rules for poor outcome or disability after stroke [55] but few have been tested against clinicians’ informal predictions [56].

### Implications for research

Although discrimination of recurrent events by clinical prediction models was poor, our study indicates that it may be similar to informal clinicians’ prediction. In addition, we identified a number of areas that could improve the discrimination of clinical prediction models for recurrent stroke or MI that future model developers could consider: (1) using all the available information from a cohort by avoiding the categorization of continuous predictors and using multiple imputation of missing data where a complete case analysis would exclude a significant proportion of the cohort; (2) reporting regression coefficients (that is, prior to any transformation) to allow more accurate evaluation of models in independent cohorts. Point score models are probably obsolete as more precise predictions can easily be obtained using applications accessed via mobile computers at the bedside. There are too many proposed models in clinical practice to remember them all, and it is only sensible that they should be available electronically; and finally, (3) measuring whether newly identified predictors (for example, blood markers or imaging techniques) add to the accurate classification of patients over more easily measured variables, for example using the net reclassification index [39,57].

A number of methodological decisions in model development may lead to clinical prediction models that make less accurate predictions [58] and we believe that an agreed set of guidelines in model development and reporting in healthcare would be helpful to developers and users of clinical prediction models alike [59].

### Limitations of the study

Assessing the quality of studies of predictive models is difficult, and there is no widely agreed set of guidelines. This is likely to become an increasing problem as such studies are frequent and very likely will begin to influence practice. Our electronic search was overly sensitive and returned a small number of relevant articles; hence, we did not perform additional searches of the ‘grey’ literature. This is an unfortunate artefact of poor indexing, as there is no Medical Subject Heading (MESH) term for clinical prediction models. We attempted to work around these limitations with forward citation searching in Google Scholar. The ESS did not classify stroke according to the Causative Classification of Stroke System (CCS); we instead manipulated a record of classification as per the Trial of Org 10172 in Acute Stroke Treatment (TOAST) algorithm to a format that closely resembled the CCS.

## Conclusions

We found that the available clinical prediction models for recurrent stroke and MI after stroke discriminated modestly between patients who do and do not have recurrent events. Clinicians’ informal predictions discriminated similarly to the models. Aspect of study design and statistical methodology were poor amongst model development studies, however, and performance might be improved with better methods.

## Abbreviations

AUROCC: area under the receiver operating characteristic curve; CI: confidence interval; EPV: events per variable; ESRS: ESSEN Stroke Risk Score; ESS: The Edinburgh Stroke Study; IQR: interquartile range; MI: myocardial infarction; PI: prediction interval; RRE-90: Recurrence Risk Estimator at 90 days; SPI-II: Stroke Prognosis Instrument II; TIA: transient ischemic attack.

## Competing interests

The authors declare that they have no competing interests.

## Authors’ contributions

WNW conceived the study. WNW, GDM and DDT contributed to the study design. WNW and DDT collected data. DDT carried out the analyses and drafted the first manuscript. WNW, MD, CLMS and GDM revised the manuscript. This study was supervised by GDM and WNW. CLMS was the principal investigator of the ESS. All authors read and approved the final manuscript.

## Pre-publication history

The pre-publication history for this paper can be accessed here:

http://www.biomedcentral.com/1741-7015/12/58/prepub

## Supplementary Material

Additional file 1 **Electronic search term implemented in Medline and EMBASE.** Further detail of included studies. Click here for file
