# Supplementary material for: Formal and informal prediction of recurrent stroke and myocardial infarction after stroke: a systematic review and evaluation of clinical prediction models in a new cohort
Source: BMC Med. 2014 Apr 4;12:58. doi: 10.1186/1741-7015-12-58 (PMC4022243; doi:10.1186/1741-7015-12-58)
Supplement: Additional file 1 — Electronic search term implemented in Medline and EMBASE. Further detail of included studies. [file 1741-7015-12-58-S1.doc]

**Supplementary data/tables/figures.**

Article title

Formal and informal prediction of recurrent stroke and myocardial infarction after stroke: a systematic review and evaluation of clinical prediction models in a new cohort

Authors

Douglas D. Thompson, Gordon D. Murray, Martin Dennis, Cathie L.M. Sudlow, William N. Whiteley

| Medline  1. cerebrovascular disorders/ or basal ganglia cerebrovascular disease/ or exp brain ischemia/ or carotid artery diseases/ or carotid artery thrombosis/ or carotid stenosis/ or cerebrovascular accident/ or exp brain infarction/ or exp hypoxia-ischemia, brain/ or exp intracranial arterial diseases/ or exp "intracranial embolism and thrombosis"/  2. ((brain or cerebr$ or cerebell$ or vertebrobasil$ or hemispher$ or intracran$ or intracerebral or infratentorial or supratentorial or middle cerebr$ or mca$ or anterior circulation) adj5 (isch?emi$ or infarct$ or thrombo$ or emboli$ or occlus$ or hypoxi$)).tw.  3. (isch?emi$ adj6 (stroke$ or apoplex$ or cerebral vasc$ or cerebrovasc$ or cva or attack$)).tw.  4. 1 or 2 or 3  5. ((risk or predictive or prediction or statistical or cox or logistic or survival or multivariate or multivariable or hazard$) and (prediction or model$ or equation or rule or calculator)).tw.  6. cox proportional hazard model$.ab. or cox proportional hazard model$.ti. or cox proportional-hazard$.ab. or cox proportional-hazard$.ti.  7. 5 or 6  8. 7 and 4  9. Child/ or ethnic groups/ or *Depression/ or economics/ or *caregivers/  10. 8 not 9  11. (Bibliography or Editorial or Letter or News).pt.  12. 10 not 11  13. limit 12 to 1980-current  14. limit 13 to human. |
| --- |
| EMBASE  1. cerebral artery disease/ or cerebrovascular accident/ or stroke/ or vertebrobasilar insufficiency/ or wallenberg syndrome/ or exp brain infarction/ or exp brain ischemia/ or exp occlusive cerebrovascular disease/ or cerbrovascular disease/ or exp carotid artery diseases/  2. ((brain or cerebr$ or cerebell$ or vertebrobasil$ or hemispher$ or intracran$ or intracerebral or infratentorial or supratentorial or middle cerebr$ or mca$ or anterior circulation) adj5 (isch?emi$ or infarct$ or thrombo$ or emboli$ or occlus$ or hypoxi$)).tw.  3. (isch?emi$ adj6 (stroke$ or apoplex$ or cerebral vasc$ or cerebrovasc$ or cva or attack$)).tw.  4. 1 or 2 or 3  5. ((risk or predictive or prediction or statistical or cox or logistic or survival or multivariate or multivariable or hazard$) and (prediction or model$ or equation or rule or calculator)).tw.  6. cox proportional hazard model$.ab. or cox proportional hazard model$.ti. or cox proportional-hazard$.ab. or cox proportional-hazard$.ti.  7. 5 or 6  8. 7 and 4  9. Child/ or ethnic group/ or depression/ or health economics/ or caregiver/  10. 8 not 9  11. (Bibliography or Editorial or Letter or News).pt.  12. 10 not 11  13. limit 12 to 1980-current  14. limit 13 to human |

Online Supplement 1 - Electronic search term implemented in Medline and EMBASE

| **Section/topic** | **#** | | **Checklist item** | **Reported on page #** |
| --- | --- | --- | --- | --- |
| **TITLE: Prediction of recurrent stroke and myocardial infarction after stroke: a systematic review of clinical prediction models** | | | |  |
| Title | 1 | | Identify the report as a systematic review, meta-analysis, or both. | Title page |
| **ABSTRACT** | | | |  |
| Structured summary | 2 | | Provide a structured summary including, as applicable: background; objectives; data sources; study eligibility criteria, participants, and interventions; study appraisal and synthesis methods; results; limitations; conclusions and implications of key findings; systematic review registration number. | Paragraph 1 |
| **INTRODUCTION** | | | |  |
| Rationale | 3 | | Describe the rationale for the review in the context of what is already known. | Paragraphs 1 and 2 |
| Objectives | 4 | | Provide an explicit statement of questions being addressed with reference to participants, interventions, comparisons, outcomes, and study design (PICOS). | Paragraph 3 |
| **METHODS** | | | |  |
| Protocol and registration | 5 | | Indicate if a review protocol exists, if and where it can be accessed (e.g., Web address), and, if available, provide registration information including registration number. | Paragraph 1 |
| Eligibility criteria | 6 | | Specify study characteristics (e.g., PICOS, length of follow-up) and report characteristics (e.g., years considered, language, publication status) used as criteria for eligibility, giving rationale. | Paragraph 2 and 3 |
| Information sources | 7 | | Describe all information sources (e.g., databases with dates of coverage, contact with study authors to identify additional studies) in the search and date last searched. | Paragraph 1 |
| Search | 8 | | Present full electronic search strategy for at least one database, including any limits used, such that it could be repeated. | Paragraph 1 and Online supplement 1 |
| Study selection | 9 | | State the process for selecting studies (i.e., screening, eligibility, included in systematic review, and, if applicable, included in the meta-analysis). | Paragraphs 2, 3 and 4 |
| Data collection process | 10 | | Describe method of data extraction from reports (e.g., piloted forms, independently, in duplicate) and any processes for obtaining and confirming data from investigators. | Paragraphs 3 and 4 |
| Data items | 11 | | List and define all variables for which data were sought (e.g., PICOS, funding sources) and any assumptions and simplifications made. | Paragraphs 3 and 4 |
| Risk of bias in individual studies | 12 | | Describe methods used for assessing risk of bias of individual studies (including specification of whether this was done at the study or outcome level), and how this information is to be used in any data synthesis. | Paragraph 5 |
| Summary measures | 13 | | State the principal summary measures (e.g., risk ratio, difference in means). | NA |
| Synthesis of results | 14 | | Describe the methods of handling data and combining results of studies, if done, including measures of consistency (e.g., I^2^) for each meta-analysis. | Paragraph 5 |
| Risk of bias across studies | 15 | | Specify any assessment of risk of bias that may affect the cumulative evidence (e.g., publication bias, selective reporting within studies). | Paragraph 5 |
| Additional analyses | 16 | | Describe methods of additional analyses (e.g., sensitivity or subgroup analyses, meta-regression), if done, indicating which were pre-specified. | NA |
| **RESULTS** | | | |  |
| Study selection | 17 | | Give numbers of studies screened, assessed for eligibility, and included in the review, with reasons for exclusions at each stage, ideally with a flow diagram. | Paragraph 1and figure 1 |
| Study characteristics | 18 | | For each study, present characteristics for which data were extracted (e.g., study size, PICOS, follow-up period) and provide the citations. | Paragraphs 2 to 7 and Online supplement 3 to 6 |
| Risk of bias within studies | 19 | | Present data on risk of bias of each study and, if available, any outcome level assessment (see item 12). | NA |
| Results of individual studies | 20 | | For all outcomes considered (benefits or harms), present, for each study: (a) simple summary data for each intervention group (b) effect estimates and confidence intervals, ideally with a forest plot. | Paragraphs 2 to 10 and figures 2 and 3 |
| Synthesis of results | 21 | | Present results of each meta-analysis done, including confidence intervals and measures of consistency. | Figure 3 |
| Risk of bias across studies | 22 | | Present results of any assessment of risk of bias across studies (see Item 15). | Online supplement 7 |
| Additional analysis | 23 | | Give results of additional analyses, if done (e.g., sensitivity or subgroup analyses, meta-regression [see Item 16]). | Paragraph 10 |
| **DISCUSSION** | | | |  |
| Summary of evidence | | 24 | Summarize the main findings including the strength of evidence for each main outcome; consider their relevance to key groups (e.g., healthcare providers, users, and policy makers). | Paragraph 1 and 4 |
| Limitations | | 25 | Discuss limitations at study and outcome level (e.g., risk of bias), and at review-level (e.g., incomplete retrieval of identified research, reporting bias). | Paragraphs 2 and 4 to 7 |
| Conclusions | | 26 | Provide a general interpretation of the results in the context of other evidence, and implications for future research. | Paragraph 5 and 8 |
| **FUNDING** | | | |  |
| Funding | | 27 | Describe sources of funding for the systematic review and other support (e.g., supply of data); role of funders for the systematic review. | NA |

Online Supplement 2 - PRISMA checklist

| Predictors | Total per article | Ay[^1^](#_ENREF_1) (RRE90) | | Dhamoon  [^2^](#_ENREF_2) | | Diener[^3^](#_ENREF_3) (ESRS) | Kamouchi[^4^](#_ENREF_4) (FSRJ) | | Kernan[^5^](#_ENREF_5) (SPI-I) | Kernan[^6^](#_ENREF_6) (SPI-II) | Pezzini[^7^](#_ENREF_7) | | | Putaala[^8^](#_ENREF_8) | | | | Stahrenberg[^9^](#_ENREF_9) | | | Sumi[^10^](#_ENREF_10) | | Suzuki[^11^](#_ENREF_11) | Wijk[^12^](#_ENREF_12) (LiLAC) | | | | | |
| --- | --- | --- | --- | --- | --- | --- | --- | --- | --- | --- | --- | --- | --- | --- | --- | --- | --- | --- | --- | --- | --- | --- | --- | --- | --- | --- | --- | --- | --- |
| **Model per publication** |  | 1 | 2 | 1 | 2 | 1 | 1 | 2 | 1 | 1 | 1 | 2 | 3 | 1 | 2 | 3 | 4 | 1 | 2 | 3 | 1 | 2 | 1 | 1 | 2 | 3 | 4 | 5 | 6 |
| **Demographics** |  |  |  |  |  |  |  |  |  |  |  |  |  |  |  |  |  |  |  |  |  |  |  |  |  |  |  |  |  |
| Age | 9 |  |  | ***** | ***** | ***** | ***** | ***** | ***** | ***** |  |  |  | ***** | ***** | ***** | ***** |  |  |  | ***** | ***** | ***** | ***** | ***** | ***** | ***** | ***** | ***** |
| Gender | 4 |  |  |  |  |  |  |  |  |  |  |  |  | ***** | ***** | ***** | ***** |  |  |  | ***** | ***** | ***** | ***** | ***** | ***** | ***** | ***** | ***** |
| **Social Factors** |  |  |  |  |  |  |  |  |  |  |  |  |  |  |  |  |  |  |  |  |  |  |  |  |  |  |  |  |  |
| Smoking | 4 |  |  |  |  | ***** | ***** | ***** |  |  |  |  |  |  | ***** | ***** | ***** |  |  |  | ***** | ***** |  |  |  |  |  |  |  |
| **Past medical history** |  |  |  |  |  |  |  |  |  |  |  |  |  |  |  |  |  |  |  |  |  |  |  |  |  |  |  |  |  |
| TIA/stroke | 7 | ***** | ***** |  |  | ***** | ***** | ***** |  | ***** |  |  |  |  | ***** |  | ***** |  |  |  | ***** | ***** | ***** |  |  |  |  |  |  |
| Stroke in family | 1 |  |  |  |  |  |  |  |  |  | ***** |  | ***** |  |  |  |  |  |  |  |  |  |  |  |  |  |  |  |  |
| CAD | 2 |  |  | ***** | ***** |  |  |  |  | ***** |  |  |  |  |  |  |  |  |  |  |  |  |  |  |  |  |  |  |  |
| PAD | 3 |  |  |  |  | ***** |  |  |  |  |  |  |  |  | ***** | ***** | ***** |  |  |  | ***** | ***** |  |  |  |  |  |  |  |
| CHF/Heart failure | 3 |  |  | ***** | ***** |  |  |  |  | ***** |  |  |  | ***** | ***** |  | ***** |  |  |  |  |  |  |  |  |  |  |  |  |
| CHD | 3 |  |  |  |  |  |  |  | ***** |  |  |  |  |  | ***** | ***** | ***** |  |  |  |  |  |  |  |  |  |  |  |  |
| MI | 5 |  |  |  | ***** | ***** |  |  |  |  |  |  |  |  | ***** |  | ***** |  |  |  | ***** | ***** |  | ***** | ***** | ***** |  |  |  |
| Diabetes | 8 |  |  |  |  | ***** | ***** | ***** | ***** | ***** |  |  |  | ***** | ***** | ***** |  |  |  |  | ***** | ***** | ***** | ***** | ***** | ***** | ***** | ***** | ***** |
| Chronic kidney disease | 1 |  |  |  |  |  | ***** | ***** |  |  |  |  |  |  |  |  |  |  |  |  |  |  |  |  |  |  |  |  |  |
| Other cardiovascular disease (not MI/AF) | 3 |  |  |  |  | ***** | ***** | ***** |  |  |  |  |  |  |  |  |  |  |  |  | ***** | ***** |  |  |  |  |  |  |  |
| Peripheral vascular surgery | 1 |  |  |  |  |  |  |  |  |  |  |  |  |  |  |  |  |  |  |  |  |  |  | ***** | ***** |  |  |  |  |
| Intermittent claudication | 1 |  |  |  |  |  |  |  |  |  |  |  |  |  |  |  |  |  |  |  |  |  |  | ***** | ***** | ***** | ***** | ***** | ***** |
| **Details of stroke** |  |  |  |  |  |  |  |  |  |  |  |  |  |  |  |  |  |  |  |  |  |  |  |  |  |  |  |  |  |
| Stroke not TIA | 3 |  |  |  |  |  |  |  | ***** | ***** |  |  |  |  |  |  |  |  |  |  |  |  |  |  | ***** | ***** |  | ***** | ***** |
| Lacunar subtype | 1 |  |  |  | ***** |  |  |  |  |  |  |  |  |  |  |  |  |  |  |  |  |  |  |  |  |  |  |  |  |
| Nonlacunar subtype | 1 |  |  |  |  |  | ***** | ***** |  |  |  |  |  |  |  |  |  |  |  |  |  |  |  |  |  |  |  |  |  |
| Embolic subtype | 1 |  |  |  | ***** |  |  |  |  |  |  |  |  |  |  |  |  |  |  |  |  |  |  |  |  |  |  |  |  |
| Subtype (not SAO) | 1 |  |  |  |  |  |  |  |  |  |  |  |  |  |  |  |  |  |  |  | ***** | ***** |  |  |  |  |  |  |  |
| Admission CCS | 2 | ***** | ***** |  |  |  |  |  |  |  |  |  |  | ***** | ***** | ***** | ***** |  |  |  |  |  |  |  |  |  |  |  |  |
| Migraine with aura | 1 |  |  |  |  |  |  |  |  |  | ***** |  | ***** |  |  |  |  |  |  |  |  |  |  |  |  |  |  |  |  |
| Amaurosis Fugax | 1 |  |  |  |  |  |  |  |  |  |  |  |  |  |  |  |  |  |  |  |  |  |  |  | ***** |  |  |  |  |
| Risk score: ESRS | 1 |  |  |  |  |  |  |  |  |  |  |  |  |  |  |  |  | ***** |  |  |  |  |  |  |  |  |  |  |  |
| Risk score: SPI-II | 1 |  |  |  |  |  |  |  |  |  |  |  |  |  |  |  |  |  | ***** |  |  |  |  |  |  |  |  |  |  |
| NIHSS | 1 |  |  |  |  |  |  |  |  |  |  |  |  |  |  |  |  |  |  | ***** |  |  |  |  |  |  |  |  |  |
| **General examination** |  |  |  |  |  |  |  |  |  |  |  |  |  |  |  |  |  |  |  |  |  |  |  |  |  |  |  |  |  |
| Waist circumference/ Obesity | 3 |  |  |  |  |  |  |  |  |  |  |  |  |  | ***** |  | ***** |  |  |  | ***** | ***** | ***** |  |  |  |  |  |  |
| Hypertension or BP | 9 |  |  |  |  | ***** | ***** | ***** | ***** | ***** | ***** |  | ***** |  | ***** | ***** | ***** |  |  |  | ***** | ***** | ***** | ***** | ***** | ***** | ***** |  |  |
| Hyperlipidemia | 1 |  |  |  |  |  |  |  |  |  |  |  |  |  |  |  |  |  |  |  |  |  | ***** |  |  |  |  |  |  |
| AF | 3 |  |  | ***** | ***** |  | ***** |  |  |  |  |  |  |  | ***** |  | ***** |  |  |  |  |  |  |  |  |  |  |  |  |
| mRS | 1 |  |  |  |  |  |  |  |  |  |  |  |  |  |  |  |  |  |  |  |  |  | ***** |  |  |  |  |  |  |
| Paresis | 1 |  |  |  |  |  |  |  |  |  |  |  |  |  |  |  |  |  |  |  |  |  |  |  |  |  |  | ***** | ***** |
| Dysarthia | 1 |  |  |  |  |  |  |  |  |  |  |  |  |  |  |  |  |  |  |  |  |  |  |  | ***** | ***** |  | ***** | ***** |
| Dyslipidemia | 1 |  |  |  |  |  |  |  |  |  |  |  |  |  | ***** |  | ***** |  |  |  |  |  |  |  |  |  |  |  |  |
| Vertigo | 1 |  |  |  |  |  |  |  |  |  |  |  |  |  |  |  |  |  |  |  |  |  |  |  | ***** | ***** |  |  |  |
| **CT/MRI/ECG** |  |  |  |  |  |  |  |  |  |  |  |  |  |  |  |  |  |  |  |  |  |  |  |  |  |  |  |  |  |
| Multiple infarcts of different ages | 1 | ***** |  |  |  |  |  |  |  |  |  |  |  |  |  |  |  |  |  |  |  |  |  |  |  |  |  |  |  |
| Simultaneous infarcts in different circulations | 1 | ***** |  |  |  |  |  |  |  |  |  |  |  |  |  |  |  |  |  |  |  |  |  |  |  |  |  |  |  |
| White matter lesions | 1 |  |  |  |  |  |  |  |  |  |  |  |  |  |  |  |  |  |  |  |  |  |  |  |  | ***** |  |  | ***** |
| Any infarct | 1 |  |  |  |  |  |  |  |  |  |  |  |  |  |  |  |  |  |  |  |  |  |  |  |  | ***** |  |  | ***** |
| Q wave on ECG | 1 |  |  |  |  |  |  |  |  |  |  |  |  |  |  |  |  |  |  |  |  |  |  |  |  | ***** |  |  |  |
| Negative T wave | 1 |  |  |  |  |  |  |  |  |  |  |  |  |  |  |  |  |  |  |  |  |  |  |  |  | ***** |  |  |  |
| ST-depression | 1 |  |  |  |  |  |  |  |  |  |  |  |  |  |  |  |  |  |  |  |  |  |  |  |  | ***** |  |  | ***** |
| **Genetic factors/biomarkers** |  |  |  |  |  |  |  |  |  |  |  |  |  |  |  |  |  |  |  |  |  |  |  |  |  |  |  |  |  |
| FV_G1691A_ | 1 |  |  |  |  |  |  |  |  |  |  | ***** | ***** |  |  |  |  |  |  |  |  |  |  |  |  |  |  |  |  |
| TT677 MTHFR | 1 |  |  |  |  |  |  |  |  |  |  | ***** | ***** |  |  |  |  |  |  |  |  |  |  |  |  |  |  |  |  |
| PT_G20210A_ | 1 |  |  |  |  |  |  |  |  |  |  | ***** | ***** |  |  |  |  |  |  |  |  |  |  |  |  |  |  |  |  |
| hsTropT | 1 |  |  |  |  |  |  |  |  |  |  |  |  |  |  |  |  | ***** | ***** | ***** |  |  |  |  |  |  |  |  |  |

Online Supplement 3 - Identified risk predictors per model. Multiple models associated with each study are denoted by individual columns. Abbreviations CAD – coronary artery disease; PAD – Peripheral Artery Disease; AF – Atrial Fibrillation; CCS – Causative Classification of Stroke System; MI – Myocardial Infarction; SAO – Small Artery Occlusion; TIA – Transient Ischaemic Attack; mRS – modified Rankin Scale

| **Study (name)** | **Baseline event** | **Derivation cohort** | **n/N** | **Outcome** | **Comment** |
| --- | --- | --- | --- | --- | --- |
| Ay[^1^](#_ENREF_1) (RRE90) | Ischaemic stroke < 72 hours of onset | Retrospectively collected, single centre hospital cohort | 60/1458 | Recurrent ischaemic stroke 90 days from baseline event | Two models: (A) a clinical-based model; and (B) a clinical- and imaging-based model. |
| Dhamoon[^2^](#_ENREF_2) | Ischaemic stroke | Prospectively recruited population based cohort | 102/655 | MI or vascular death 5 years from baseline event | Two models developed; ethnically diverse population |
| Diener[^3^](#_ENREF_3) (ESRS) | Ischaemic stroke within ≥1 week & ≤6months | Multicentre randomised clinical trial (CAPRIE) | NA/6431 | Stroke recurrence 1 year from baseline event | Not well described. Appears in a number of different papers |
| Kamouchi[^4^](#_ENREF_4) (FSRJ) | Ischaemic stroke within 7 days | Multicentre, hospital based, prospective and reterospective registry | 175/3067 | Recurrent ischaemic stroke 1 year from baseline event | Developed for specific use in Japanese populations |
| Kernan[^5^](#_ENREF_5) (SPI-I) | TIA or minor stroke | Retrospectively collected, single hospital cohort | 38/142 | Stroke or death 2 years from baseline event |  |
| Kernan[^6^](#_ENREF_6) (SPI-II) | TIA or minor stroke <90days of onset | Randomised clinical trial (WEST) | 90/525 | Risk of recurrent stroke or death 2 years from baseline event | A modification of SPI-I. Additional predictors selected using an all-female cohort |
| Pezzini[^7^](#_ENREF_7) | First ever ischaemic stroke aged 18-45 | Prospective hospital based cohort | 73/511 | Risk of ischaemic recurrence: fatal/nonfatal MI, IS, OR TIA 4 years from baseline event | Three models: non-genetic; genetic score; and both. Derived in a young adult population. |
| Putaala[^8^](#_ENREF_8) | First ever ischaemic stroke aged 15-49 | Prospective hospital based cohort | 72/807 | Fatal/nonfatal IS. OR the composite outcome: fatal/nonfatal stroke OR MI 5 years from baseline event | Four models developed. Derived in a young adult population. |
| Stahrenberg[^9^](#_ENREF_9) | Ischaemic stroke | Prospective observational trial | 23/197 | Cardiovascular events one year from baseline | Modification of the ESRS and the SPI-II. Six models suggested using a blood biomarker |
| Sumi[^10^](#_ENREF_10) | Ischaemic stroke | Large prospective registry | 133/3290 | Ischaemic stroke or cardiovascular events 1 year from baseline event | Modification of the ESRS: two models, one for IS and one for cardiovascular events |
|  |  |  |  |  |  |
|  |  |  |  |  |  |
| Suzuki[^11^](#_ENREF_11) | Ischaemic stroke 2weeks – 6months | Multiple institutions representing all of Japan | NA/3324 | Recurrent ischaemic stroke |  |
| Wijk[^12^](#_ENREF_12) (LiLAC) | TIA or minor stroke | Randomised trial Dutch TIA Trial with retrospectively collected events | NA/2362 | Long-term vascular event risk 10 years from baseline event | Six models developed with increasing complexity |

Online Supplement 4 - Models for predicting risk of vascular events

| Study | Model | Cohort | Baseline event | Endpoint used | Country | Follow-up | Recruitment Period (MM/YYYY) | Comment |
| --- | --- | --- | --- | --- | --- | --- | --- | --- |
| Alvarez-Sabin[^13^](#_ENREF_13) | ESRS | 88 hospitals | IS | Stroke OR new vascular events | Spain | 6 months | 01/2005 – 06/2005 | Only reported sensitivity and specificity. Suggests an improvement to the ESRS adding alcoholism and changing the cut-point |
| Ay[^1^](#_ENREF_1) | ESRS and SPI-II | Single centre | IS | Stroke | America | 90 days | 2003 - 2006 | Substantially lower follow-up period than used in original model development, 14 days |
| Chandratheva[^14^](#_ENREF_14) | ESRS, SPI-II and ABCD2 | Population based study; 63 GPs | TIA or Stroke | Stroke | Oxfordshire, UK | 90 days | 04/2002 – 03/2007 | ESRS>2 as high risk, SPI-II split into [0-3], [4-7] and [8-15]. Authors also made a 7 days assessment. Supporting evidence that ABCD2may be applied to TIA or stroke. |
| Fittzek[^15^](#_ENREF_15) | ESRS | Stroke Unit | TIA or acute IS | Stroke | Germany | Mean: 13.4 months, SD: 5.9 | 01/2007 – 09/2008 | ESRS>2 as high risk, although they suggest ESRS>3 is better, and that the inclusion of more variables may help |
| Kernan[^6^](#_ENREF_6) | SPI-II | Trial data | TIA or IS | Stroke OR death | NA | 2 years | NA | Pooled AUROCC from three individual sets of trial data (UK-TIA, CAPRIE, NoMaSS) refer to original paper for sample size in each |
| Maier[^16^](#_ENREF_16) | ESRS, ABCD2 and RRE-90 | Stroke Unit | IS | Stroke OR CV death | Germany | Median: 8 days, (IQR 6 to 11) | 2007 – 2011 | Supporting evidence that ABCD2 may be applied to TIA or stroke. |
| Meng[^17^](#_ENREF_17) | ESRS and SPI-II | Register of 132 hospitals | TIA or IS | Stroke AND combined vascular event | China | 1 year | 09/2007 – 08/2008 | Admitted within 14 days from onset |
| Navi[^18^](#_ENREF_18) | SPI-II | Community based | All severities of IS | IS AND/OR death | N. California | 1 year following discharge | 2004 – 2006 | Stroke recurrence in isolation is shown to be less well predicted by SPI-II |
| Stahrenberg[^9^](#_ENREF_9) | ESRS and SPI-II | Prospective observational trial | IS | Cardiovascular events OR total mortality | Germany | 1 year | 03/2009 – 02/2010 | Investigated the added benefit of blood biomarkers. Demonstrated a significant net improvement by adding high sensitivity troponin |
| Sumi[^10^](#_ENREF_10) | ESRS | Large prospective registry | IS | Stroke OR MI OR cardiovascular death | Japan | 1 year | 01/2007 – 05/2008 | 313 GP sites. Authors suggest a modified ESRS |
| Weimar[^19^](#_ENREF_19) | ESRS, SPI-II and LiLAC | 10 Stroke centres | TIA or non-disabling Stroke | Stroke, OR stroke AND cardiovascular death | Germany | Median: 1 year | 08/2005 – 12/2006 | Baseline event had mRS<4, recommended cut-offs for ESRS and SPI-II were used. Patients without follow-up were significantly older |
| Weimar[^20^](#_ENREF_20) | ESRS | 85 Stroke Units | TIA or acute IS | Stroke, OR stroke AND cardiovascular death | Germany | Mean: 17.5 months, SD: 0.88 | 07/2005 – 10/2005 | Authors also assessed the discriminative ability of the *Ankle Brachial Index* |
| Weimar[^21^](#_ENREF_21) | ESRS | Observational registry | TIA or IS | Non-fatal stroke, nonfatal MI, and cardiovascular death | REACH Registry | 1 year | 12/2003 – 06/2004 | Representative of stable cerebrovascular patients worldwide, but under represents African and Chinese populations |
|  |  |  |  |  |  |  |  |  |
|  |  |  |  |  |  |  |  |  |
| Weimar[^22^](#_ENREF_22) | ESRS and SPI-II | 15 rehabilitation centres | IS | Non-fatal stroke, nonfatal MI, and cardiovascular death | Germany | 1 year | 05/2008 – 09/2008 | Mean delay from stroke onset 0.9 ± 0.5 months. Index event occurred <3 months before entry |
| Wijnhound[^23^](#_ENREF_23) | SPI-II | Single hospital | TIA or minor IS | Stroke, MI AND vascular death OR fatal/non-fatal stroke | Netherlands | 2 years | NA | Proposed improvement: use of continuous variables instead of categorised |

Online Supplement 5 - Evaluation studies for ESRS, SPI-II and LiLAC. Abbreviations: TIA – Transient ischaemic attack; IS – ischaemic stroke; ESRS – Essen Stroke Risk Score; SPI-II – Stroke Prognosis Instrument II; LiLAC – Life Long after Cerebral ischemia; SD – standard deviation; mRS – modified Rankin Scale

| **Model** | **Study** | **AUROCC** | **95% CI** | **Size (n/N)** | **Outcome** | **Additional AUROCC with 95% CI** |
| --- | --- | --- | --- | --- | --- | --- |
|  |  |  |  |  |  |  |
| **ESRS** | Ay[^1^](#_ENREF_1) | 0.59 | (0.53, 0.66) | NA | Ischaemic stroke | - |
|  | Chandretheva[^14^](#_ENREF_14) | 0.50 | (0.42, 0.59) | 49/520 | Stroke | 0.49 (0.35, 0.62), for 7 day events |
|  | Fitzek[^15^](#_ENREF_15) | 0.59 | NA | 76/723 | Stroke | - |
|  | Weimar[^19^](#_ENREF_19) | 0.65 | (0.60, 0.69) | 135/1897 | Stroke or CV death | 0.62 (0.57, 0.67) for stroke |
|  | Weimar[^20^](#_ENREF_20) | 0.61 | (0.54, 0.69) | 60/700 | Stroke or CV death | 0.56 95%CI. NA for stroke |
|  | Weimar[^21^](#_ENREF_21) | 0.60 | (0.58, 0.62) | NA/15605 | Nonfatal stroke/MI or CV death | 0.56 (0.53, 0.58) for stroke |
|  | Weimar[^22^](#_ENREF_22) | 0.59 | (0.56, 0.63) | NA/846 | Nonfatal stroke/MI or CV death | 0.62 (0.59, 0.65) fatal/non-fatal stroke |
|  | Maier[^16^](#_ENREF_16) | 0.59 | (0.50, 0.68) | 95/1727 | Stroke | 0.50 (0.42, 0.58), for 7 day events |
|  | Meng[^17^](#_ENREF_17) | 0.60 | (0.59, 0.61) | NA/11384 | Nonfatal stroke/MI or CV death | 0.59 (0.58, 0.60) for stroke, for baseline IS only: 0.60 (0.59, 0.62) for nonfatal stroke/MI or CV death and 0.60 (0.57, 0.61) for stroke |
|  | Stahrenberg[^9^](#_ENREF_9) | 0.695 | (0.567, 0.822) | 23/197 | Stroke or CV death | 0.744 (0.575, 0.912), for all-cause mortality |
|  | Sumi[^10^](#_ENREF_10) | 0.613 | (0.564, 0.661) | 133/3292 | Stroke/MI or CV death | 0.604 (0.554, 0.654) for stroke |
|  |  |  |  |  |  |  |
| **SPI-II** | Ay[^1^](#_ENREF_1) | 0.56 | (0.49, 0.64) | NA | Ischaemic stroke | - |
|  | Chandretheva[^14^](#_ENREF_14) | 0.48 | (0.39, 0.60) | 49/514 | Stroke | 0.50 (0.37, 0.64), for 7 day events |
|  | Kernan (2000)[^6^](#_ENREF_6) | 0.63 | (0.62, 0.65) | 1241/9220 | Stroke or death | - |
|  | Navi[^18^](#_ENREF_18) | 0.62 | (0.61, 0.64) | 1422/5575 | Stroke or death | 0.55 (0.51, 0.59) stroke; 0.64 (0.62, 0.66) death |
|  | Weimar[^19^](#_ENREF_19) | 0.66 | (0.61, 0.70) | 135/1897 | Stroke or CV death | 0.65 (0.60, 0.70) for stroke |
|  | Weimar[^22^](#_ENREF_22) | 0.60 | (0.57, 0.64) | NA/846 | Nonfatal stroke/MI or CV death | 0.56 (0.53, 0.60) fatal/non-fatal stroke |
|  | Wijnhound[^23^](#_ENREF_23) | 0.68 | (0.61, 0.75) | 57/592 | Stroke/MI or CV death | 0.64 (0.56, 0.72) fatal/non-fatal stroke |
|  | Meng[^17^](#_ENREF_17) | 0.60 | (0.58, 0.61) | NA/11384 | Nonfatal stroke/MI or CV death | 0.59 (0.58, 0.61) for stroke, for baseline IS only: 0.61 (0.59, 0.62) for nonfatal stroke/MI or CV death and 0.60 (0.58, 0.62) for stroke |
|  | Stahrenberg[^9^](#_ENREF_9) | 0.699 | (0.587, 0.810) | 23/197 | Stroke or CV death | 0.708 (0.549, 0.867), for all-cause mortality |
|  |  |  |  |  |  |  |
| **ABCD2** | Chandretheva[^14^](#_ENREF_14) | 0.62 | (0.54, 0.70) | 49/520 | Stroke | 0.64 (0.53, 0.74), for 7 day events |
|  | Maier[^16^](#_ENREF_16) | 0.60 | (0.52, 0.69) | 95/1727 | Stroke | 0.60 (0.53, 0.67), for 7 day events |
|  |  |  |  |  |  |  |
| **LiLAC** | Weimar[^19^](#_ENREF_19) | 0.65 | (0.61, 0.70) | 135/1897 | Stroke or CV death | 0.64 (0.59, 0.69) for stroke |
|  |  |  |  |  |  |  |
|  |  |  |  |  |  |  |
|  |  |  |  |  |  |  |
|  |  |  |  |  |  |  |
| **RRE-90 (Model A)** | Maier[^16^](#_ENREF_16) | 0.72 | (0.56, 0.88) | 95/1727 | Stroke | 0.58 (0.46, 0.70), for 7 day events |
| **RRE-90 (Model B)** | Maier | 0.75 | (0.66, 0.84) | 95/1727 | Stroke | 0.68 (0.58, 0.77), for 7 day events |

Online Supplement 6 - Discrimination metrics for externally evaluated models (n = number of events, N = total sample size, CV = cardiovascular)

Online Supplement 7 - Contour-enhanced funnel plots for assessing publication bias in ESRS and SPI-II evaluation studies

| Sensitivity analyses | Number of associated studies | AUROCC | 95% CI | 95% PI |
| --- | --- | --- | --- | --- |
| All studies |  |  |  |  |
| ESRS[^3^](#_ENREF_3) | 10 | 0.60 | 0.59 to 0.62 | 0.57 to 0.63 |
| SPI-II[^6^](#_ENREF_6) | 9 | 0.62 | 0.60 to 0.64 | 0.56 to 0.67 |
| Outcome as per development |  |  |  |  |
| ESRS[^3^](#_ENREF_3) | 8 | 0.59 | 0.57 to 0.61 | 0.28 to 0.91 |
| SPI-II[^6^](#_ENREF_6) | 7 | 0.62 | 0.61 to 0.64 | 0.39 to 0.86 |
| Follow-up time as per development |  |  |  |  |
| ESRS[^3^](#_ENREF_3) | 5 | 0.61 | 0.59 to 0.62 | 0.43 to 0.78 |
| SPI-II[^6^](#_ENREF_6) | 1 | 0.68 | 0.61 to 0.75 | NA |
| Follow-up and outcome as per development |  |  |  |  |
| ESRS[^3^](#_ENREF_3) | 4 | 0.60 | 0.57 to 0.63 | 0.24 to 0.95 |
| SPI-II[^6^](#_ENREF_6) | 1 | 0.68 | 0.61 to 0.75 | NA |

Online Supplement 8 - Sensitivity analyses of published AUROCC values for the ESRS and SPI-II models (note on two occasions a meta-analysis of SPI-II was not possible)

| **Variable** | **All (N=1257)** | **No. Missing** |  | **Outpatients (N=671)** | **No. Missing** |
| --- | --- | --- | --- | --- | --- |
| **Doctor’s experience** |  |  |  |  |  |
| Fully trained vs in training, n (%)^1^ | 653 (52) | 155 (12) |  | 444 (66) | 130 (19) |
| Stroke specialist vs neurologist, n (%)^1^ | 755 (60) | 155 (12) |  | 387 (58) | 130 (19) |
| **Baseline characteristics** |  |  |  |  |  |
| Age | 74 (65 to 81) |  |  | 71 (62 to78) | - |
| < 65 years | 26% (321) | - |  | 30% (203) | - |
| 65 – 75 years | 46% (577) | - |  | 37% (247) | - |
| > 75 years | 29% (359) | - |  | 33% (221) | - |
| > 70 years vs. ≤70 | 61% (770) | - |  | 55% (367) | - |
| Male | 51% (644) | - |  | 55% (368) | - |
| History of hypertension | 54% (683) | 1 |  | 53% (354) | - |
| History of diabetes mellitus | 13% (158) | - |  | 12% (82) | - |
| Previous MI | 28% (350) | - |  | 23% (157) | - |
| Heart Failure | 6% (80) | 3 |  | 3% (19) | 2 |
| Current or prior atrial fibrillation | 22% (271) | 2 |  | 13% (90) | 3 |
| PAD | 8% (96) | 5 |  | 9% (63) | 1 |
| Current or Ex-Smoker < 12 months | 66% (820) | 22 |  | 70% (463) | 5 |
| Additional TIA or ischaemic stroke (excluding qualifying event) | 31% (391) | 3 |  | 27% (180) |  |
| Admission CCS subtype |  |  |  |  |  |
| Large Artery Atherosclerosis | 8% (104) | - |  | 7% (49) | **-** |
| Cardio-aortic embolism | 14% (171) | - |  | 10% (64) | **-** |
| Small Artery occlusion | 21% (258) | - |  | 27% (179) | **-** |
| Other Causes | 8% (105) | - |  | 7% (44) | **-** |
| Undetermined Causes | 49% (619) | - |  | 50% (335) | **-** |
| **Outcomes** |  |  |  |  |  |
| Recurrent stroke |  |  |  |  |  |
| Within 90 days | 4% (52) | - |  | 3% (20) | - |
| Within one year | 8% (102) | - |  | 7% (50) | - |
| Myocardial infarction within one year | 2% (28) | - |  | 1% (9) | - |
| Any vascular event within one year | 22% (274) | - |  | 12% (80) | - |

Online Supplement 9 Baseline characteristics for Edinburgh Stroke Study in those with ischaemic stroke on admission. NOTE: 1 – amongst outpatients where vascular prediction was available

References

1. Ay H, Gungor L, Arsava EM, Rosand J, Vangel M, Benner T, et al. A score to predict early risk of recurrence after ischemic stroke. *Neurology* 2010;74(2):128-35.

2. Dhamoon MS, Tai W, Boden-Albala B, Rundek T, Paik MC, Sacco RL, et al. Risk of myocardial infarction or vascular death after first ischemic stroke: The northern Manhattan study. *Stroke* 2007;38(6):1752-58.

3. Diener H-C, Ringleb PA, Savi P. Clopidogrel for the secondary prevention of stroke. *Expert Opinion on Pharmacotherapy* 2005;6(5):755-64.

4. Kamouchi M, Kumagai N, Okada Y, Origasa H, Yamaguchi T, Kitazono T. Risk Score for Predicting Recurrence in Patients with Ischemic Stroke: The Fukuoka Stroke Risk Score for Japanese. *Cerebrovascular Diseases* 2012;34(5-6):351-57.

5. Kernan WN, Horwitz RI, Brass LM, Viscoli CM, Taylor KJW. A Prognostic System for Transient Ischemia or Minor Stroke. *Annals of Internal Medicine* 1991;114(7):552-57.

6. Kernan WN, Viscoli CM, Brass LM, Makuch RW, Sarrel PM, Roberts RS, et al. The stroke prognosis instrument II (SPI-II): A clinical prediction instrument for patients with transient ischemia and nondisabling ischemic stroke. *Stroke* 2000;31(2):456-62.

7. Pezzini A, Grassi M, Del Zotto E, Lodigiani C, Ferrazzi P, Spalloni A, et al. Common genetic markers and prediction of recurrent events after ischemic stroke in young adults. *Neurology* 2009;73(9):717-23.

8. Putaala J, Haapaniemi E, Metso AJ, Metso TM, Artto V, Kaste M, et al. Recurrent ischemic events in young adults after first-ever ischemic stroke. *Annals of Neurology* 2010;68(5):661-71.

9. Stahrenberg R, Niehaus C-F, Edelmann F, Mende M, Wohlfahrt J, Wasser K, et al. High-sensitivity troponin assay improves prediction of cardiovascular risk in patients with cerebral ischaemia. *J Neurol Neurosurg Psychiatry* 2013.

10. Sumi S, Origasa H, Houkin K, Terayama Y, Uchiyama S, Daida H, et al. A modified Essen stroke risk score for predicting recurrent cardiovascular events: development and validation. *International Journal of Stroke* 2012:n/a-n/a.

11. Suzuki N, Sato M, Houkin K, Terayama Y, Uchiyama S, Daida H, et al. One-Year Atherothrombotic Vascular Events Rates in Outpatients with Recent Non-Cardioembolic Ischemic Stroke: The EVEREST (Effective Vascular Event REduction after STroke) Registry. *Journal of Stroke and Cerebrovascular Diseases* 2012;21(4):245-53.

12. van Wijk I, Kappelle LJ, van Gijn J, Koudstaal PJ, Franke CL, Vermeulen M, et al. Long-term survival and vascular event risk after transient ischaemic attack or minor ischaemic stroke: a cohort study. *The Lancet* 2005;365(9477):2098-104.

13. Alvarez-Sabin J, Quintana M, Rodriguez M, Arboix A, Ramirez JM, Fuentes B. [Validation of the Essen risk scale and its adaptation to the Spanish population. Modified Essen risk scale]. *Neurologia* 2008;23(4):209-14.

14. Chandratheva A, Geraghty OC, Rothwell PM. Poor performance of current prognostic scores for early risk of recurrence after minor stroke. *Stroke* 2011;42(3):632-37.

15. Fitzek S, Leistritz L, Witte OW, Heuschmann PU, Fitzek C. The Essen Stroke Risk Score in One-Year Follow-Up Acute Ischemic Stroke Patients. *Cerebrovascular Diseases* 2011;31(4):400-07.

16. Maier IL, Bauerle M, Kermer P, Helms HJ, Buettner T. Risk prediction of very early recurrence, death and progression after acute ischaemic stroke. *European Journal of Neurology* 2013;20(4):599-604.

17. Meng X, Wang Y, Zhao X, Wang C, Li H, Liu L, et al. Validation of the Essen Stroke Risk Score and the Stroke Prognosis Instrument II in Chinese Patients. *Stroke* 2011;42(12):3619-20.

18. Navi BB, Kamel H, Sidney S, Klingman JG, Nguyen-Huynh MN, Johnston SC. Validation of the stroke Prognostic Instrument-II in a large, modern, community-based cohort of ischemic stroke survivors. *Stroke* 2011;42(12):3392-96.

19. Weimar C, Benemann J, Michalski D, Muller M, Luckner K, Katsarava Z, et al. Prediction of recurrent stroke and vascular death in patients with transient ischemic attack or nondisabling stroke: a prospective comparison of validated prognostic scores. *Stroke* 2010;41(3):487-93.

20. Weimar C, Goertler M, Rother J, Ringelstein EB, Darius H, Nabavi DG, et al. Predictive value of the Essen Stroke Risk Score and Ankle Brachial Index in acute ischaemic stroke patients from 85 German stroke units. *J Neurol Neurosurg Psychiatry* 2008;79(12):1339-43.

21. Weimar C, Diener H-C, Alberts MJ, Steg PG, Bhatt DL, Wilson PWF, et al. The Essen Stroke Risk Score Predicts Recurrent Cardiovascular Events. *Stroke* 2009;40(2):350-54.

22. Weimar C, Siebler M, Brandt T, Römer D, Rosin L, Bramlage P, et al. Vascular risk prediction in ischemic stroke patients undergoing in-patient rehabilitation – insights from the investigation of patients with ischemic stroke in neurologic rehabilitation (INSIGHT) registry. *International Journal of Stroke* 2012:n/a-n/a.

23. Wijnhoud AD, Maasland L, Lingsma HF, Steyerberg EW, Koudstaal PJ, Dippel DWJ. Prediction of Major Vascular Events in Patients With Transient Ischemic Attack or Ischemic Stroke. *Stroke* 2010;41(10):2178-85.
